# Assessment of semen quality in pure and crossbred Jersey bulls

**DOI:** 10.14202/vetworld.2015.1266-1272

**Published:** 2015-10-31

**Authors:** Umesh Kumar, Ajay P. Gawande, Sunil K. Sahatpure, Manoj S. Patil, Chetan K. Lakde, Sachin W. Bonde, Pradnyankur L. Borkar, Ajay J. Poharkar, Baldeo R. Ramteke

**Affiliations:** 1 Department of Animal Reproduction Gynaecology & Obstetrics, Nagpur Veterinary College, Maharashtra Animal and Fishery Sciences University, Nagpur, Maharashtra, India; 2 Department of Veterinary Biochemistry, Nagpur Veterinary College, Maharashtra Animal and Fishery Sciences University, Nagpur, Maharashtra, India; 3 Frozen Semen Laboratory, Nagpur, Maharashtra, India

**Keywords:** acridine orange, acrosomal integrity, crossbred Jersey, DNA integrity, Jersey, semen

## Abstract

**Aim:**

To compare the seminal attributes of neat, pre-freeze (at equilibration), and post-freeze (24 h after freezing) semen in pure and crossbred Jersey bulls.

**Materials and Methods:**

Total 36 ejaculates (3 ejaculates from each bull) were collected from 6 pure Jersey and 6 crossbred Jersey bulls and evaluated for various seminal attributes during neat, pre-freeze, and post-freeze semen.

**Results:**

The mean (±standard error [SE]) values of neat semen characteristics in pure and crossbred Jersey bulls were recorded such as volume (ml), color, consistency, mass activity (scale: 0-5), and sperm concentration (millions/ml). The extended semen was further investigated at pre-freeze and post-freeze stages and the mean (±SE) values recorded at neat, pre-freeze, and post-freeze semen were compared between pure and crossbred Jersey bulls; sperm motility (80.55±1.70%, 62.77±1.35%, 46.11±1.43% vs. 80.00±1.80%, 65.00±1.66%, 47.22±1.08%), live sperm count (83.63±1.08%, 71.72±1.09%, 58.67±1.02% vs. 80.00±1.08%, 67.91±1.20%, 51.63±0.97%), total abnormal sperm count (8.38±0.32%, 12.30±0.39%, 16.75±0.42% vs. 9.00±0.45%, 12.19±0.48%, 18.11±0.64%), hypo-osmotic swelling (HOS) reacted spermatozoa (71.88±0.77%, 62.05±0.80%, 47.27±1.05% vs. 72.77±1.02%, 62.11±0.89%, 45.94±1.33%), acrosome integrity (89.05±0.83%, 81.33±0.71%, 71.94±0.86% vs. 86.55±0.57%, 78.66±0.42%, 69.38±0.53%), and DNA integrity (99.88±0.07%, 100, 99.66±0.11% vs. 99.94±0.05%, 100, 99.44±0.18%,). The volume, color, consistency, sperm concentration, and initial motility in pure and crossbred Jersey bulls did not differ significantly (p>0.05). The mass activity was significantly (p<0.05) higher in pure Jersey as compare to crossbred Jersey bulls. Live sperm percentage and acrosome integrity was significantly (p<0.01) higher in pure Jersey bulls as compared to crossbred Jersey bulls. However, no statistical difference (p>0.05) was observed in abnormal sperm; HOS reacted spermatozoa and DNA integrity percentage among breeds.

**Conclusion:**

It may be concluded that the quality of pure Jersey bull semen was comparatively better than the crossbred Jersey bulls.

## Introduction

A dairy cow is the backbone of the Indian dairy industry as it contributes about 38% of the national [1] and 8.7% of the world total cow milk production (FAO, 2012). FAO [2] had reported that there were 620 million dairy cows in the world, out of which India had a total of 54 million (8.7% of world production). The Jersey breed of dairy cattle originated on the Island of Jersey, a small British Island located in the English Channel off the coast of France. The ability of Jerseys to make more profit per acre is one of the most important reasons for the breed’s popularity. The breed’s greater efficiency in converting feed into milk and the ability of the dairymen to keep more animals on an acre of ground are principal reasons why Jerseys make more profit per acre. They cost less to raise than other dairy cattle because they reach a productive age from 2 to 10 months earlier than other dairy breeds. They are especially tolerant to hot temperature, yet perform well in the northern climates.

However, the genetic improvement of the Indian cow was not good. The genetic improvement of dairy cow done by maximum utilization of elite sires. Reproductive efficiency of dairy cattle production can be improved through utilization of highly fertile bull bearing excellent semen quality. The effective screening methods for ejaculates prior to processing are necessary for improving reproductive performance in bulls. Certain *in vitro* tests on semen have been performed to examine structural characteristics and to observe crucial aspect of sperm function to detect the ability of sperm. The sperm function tests such as evaluation of plasma membrane integrity, determination of acrosomal status, and investigation of DNA integrity are finding their place in the modern spermatological laboratory which should be routinely applied to the evaluation of commercially produced bull semen to increase the conception rate of dairy cow. Semen quality tests are important snapshots at a particular point of time. So, it is mandatory to do an evaluation of semen, either fresh or frozen-thawed to predict its fertility indices. Semen volume, concentration of spermatozoa, proportion of dead and abnormal spermatozoa, and motility of spermatozoa are recognized as important indices of semen quality and significantly correlated with freezability and/or fertility of bovine semen [3]. Sires contribute 50% of the genetic of the offspring. A bull has a larger impact on the herd productivity than a single female: A common saying is the bull is half of the herd. When artificial insemination (AI) is applied; each bull can serve thousands of female per year. The success of AI depends on the ability to screen for semen with high fertilization potential [4]. Therefore, is extremely important to do the selection of breeding bull to improve their offspring.

So, the objective of the present study was to comparison between the reproductive performance of pure Jersey and their crossbred bulls.

## Materials and Methods

### Ethical approval

Ethical approval was taken from the Maharashtra Livestock Development Board (MLDB), Maharashtra as Frozen Semen Laboratory of Nagpur come under this organization and the experiment followed the guidelines of Institutional Animal Ethics Committee and experiment were conducted in such a way that cruelty to the animals was minimized to the minimum.

### Semen sampling

Twelve healthy bulls (6 pure Jersey and 6 crossbred Jersey bulls) belonged to Frozen Semen Laboratory, Nagpur were selected having age ranging from 6 to 8 years. Total 36 ejaculates (3 ejaculates from each bull) were collected and evaluated for various seminal attributes. Semen was collected from these bulls twice a week with an artificial vagina (40 cm long and 6.5 cm in diameter) maintained at 42-45°C. Immediately after collection, semen was first observed for macroscopic characteristics *viz*. volume, color, and consistency and then maintained at 37°C in water bath for further microscopic evaluation (mass activity, initial motility, live sperm per cent, total abnormal sperm percent). The functional ability of spermatozoa was assessed by subjecting the sperm to the test *viz*. hypo-osmotic swelling test (HOST), intact acrosome and DNA integrity. The semen was diluted with tris egg yolk-glycerol. Subsequently, semen was evaluated at pre-freeze (at equilibrated) and post-freeze (24 h after freezing) stage.

### HOST

Two hypo-osmotic solutions were prepared as follows: 2.7% aqueous solution fructose (1.351 g/50 ml distilled water) and 1.47% aqueous solution of sodium citrate (0.735/50 ml distilled water). Equal volumes of both solutions (0.5 ml each) were mixed and kept in an incubator at 37°C for 10 min. 50 µl of semen was added in above hypo-osmotic solution (fructose + sodium citrate) and incubated at 37°C for 30 min. After incubation mix, the contest gently and add 0.1 ml of buffered formal saline mix gently. 10 µl of this mixture was taken on a glass slide and covered with glass cover. The slide was observed under ×10 objective lens to determine the number of spermatozoa showing swollen head and coiled tail indicating sperms with the intact plasma membrane (HOS positive sperm). A Total 100 spermatozoa were counted to determine the percentage of HOS positive spermatozoa.

### Test for DNA damage

Assessment of DNA damage was carried out by acridine orange test. The stock solution was prepared by 0.1 g of acridine orange (from Sigma, Germany) with 100 ml of distilled water and stored in dark. The working solution was then prepared by mixing 10 ml of stock solution with 40 ml 0.1 M citric acid and then finally mixed with 2.5 ml of 0.3 M disodium hydrogen phosphate. The working stock solution prepared by 5.234 g citric acid dissolve in 250 ml distilled water and 4.06 g disodium hydrogen phosphate dissolve in 100 ml of distilled water. Kept the solution in dark and cool place. A thin smear of semen (neat/pre/post) was prepared. The smear was fixed in Carnoy’s solution (3:1, Methanol-Glacial acetic acid) for 14 h at 4°C. The slide was died for a few minutes and working solution was layered (2-3 ml) over the slide and kept for 5 min at dark and cool place. The slide was rinsed in water and covered with a cover slip. The slide was read on a fluorescence microscope using 490 nm excitation filter and 530 nm barrier filter. The total of 100 sperms were counted (red and green) and the average was calculated. Green sperms indicated sperms with intact (non-damaged) DNA and red/yellow sperm indicated sperm with damaged DNA.

### Semen evaluation

The volume of semen was recorded directly from the graduated semen collection tube immediately after collection [5]. Color of semen was graded as watery/opalescent, milky white, yellowish, and creamy [6]. Consistency was recorded as watery, milky, thin creamy, creamy, and thick creamy [7]. The mass activity was graded as described by Herman *et al*. [8]. The concentration of spermatozoa (million/ml) in the neat semen was determined by the bovine photometer No. 1062 (Photometer, IMV Technologies, France). Percentage of live spermatozoa was estimated by differential staining technique using Eosin-Nigrosin stain [9], and percentage of abnormal spermatozoa was estimated standard procedure [10]. Acrosome integrity was assessed by Giemsa staining as per Watson [11], HOST was done as per the Jeyendran *et al*. [12] and assessment of DNA damage was carried out by Acridine orange test as describe by Tejada *et al*. [13].

### Statistical analysis

The data were analyzed by the descriptive method, Student’s *t*-test (used to compare between two different breeds), and ANOVA (to compare seminal attributes of the two breeds), and result was presented as mean±standard error [14].

## Results and Discussion

### Volume

The mean volume recorded for pure and crossbred Jersey bulls were 6.50±0.39 and 5.98±0.34, respectively with no significant difference (P>0.05) between breeds (Table-1). The semen volume recorded in pure Jersey bulls was in agreement with Nilani *et al*. [15]. The semen volume recorded in crossbred Jersey bull by Sugulle *et al*. [16] and Giri *et al*. [17] found to be more or less similar with the present findings.

**Table-1:** Neat semen characteristics in pure and crossbred Jersey bulls.

Seminal characters	Mean±SE (n=18)		t-statistics

	Pure Jersey	Crossbred Jersey	
Volume	6.50±0.39	5.98±0.34	0.99
Color	Creamy white	Creamy white	-
Mass activity	4.00±0.16	3.50±0.12	2.47*
Sperm concentration (millions/ml)	1171.01±56.09	1093.488±48.25	1.047

* Significant at 5%. SE=Standard error

### Color

The color of semen was recorded as creamy white in almost all samples of both breeds (Table-1) which was similar to be observed by Tan *et al*. [18]. Few ejaculates of bulls in the present study produced yellowish semen which might be due to the presence of riboflavin in seminal plasma [19].

### Mass activity

The mass activity of pure Jersey bulls (4.00±0.16) was found to be significantly (P<0.05) higher than (3.50±0.12) crossbred Jersey bulls (Table-1). The mass activity of pure Jersey bulls was comparable with the observation recorded by Kohli [20]. The finding in crossbred Jersey bulls was in corroborates with the observations of Gupta *et al*. [21]. However, Suryaprakasam and Narasimha Rao [22] reported mass activity of semen in crossbred Jersey bulls which was lower than the present findings. A significant difference in mass activity recorded in the present study may be due to variations in degree of sexual excitement and age of bulls [23] and breed characteristics.

### Sperm concentration

The average sperm concentration in pure and crossbred Jersey bulls were 1171.01±56.09 and 1093.488±48.25 million/ml which did not differ significantly (P>0.05) (Table-1).

### Initial motility

Initial sperm motility of neat semen in pure and crossbred Jersey bulls was 80.55±1.70 and 80.00±1.80% which did not differ significantly (p>0.05) between the breeds (Table-2). The motility recorded after equilibration decreased significantly (p<0.05) from its initial stage in both breeds but did not differ significantly between breeds (Table-2). The sperm motility at post-freeze stage reduced significantly (p<0.01) from its pre-freeze and neat semen stage in both breeds but did not differ significantly between two breeds (Table-2). The motility of sperm decreased from neat to post-freeze semen in present research work was may be due to the acids arising from anerobic sperm metabolism inhibiting the sperm movement [24]. Furthermore, motility also decreases due to the cooling, freezing, and thawing procedure.

**Table-2:** Mean of seminal attributes in pure and crossbred Jersey bulls at neat, pre-freeze, and post-freeze stages (by ANOVA – oneway pathway).

Semen characteristics	Breeds	Neat semen	Pre-freeze	Post-freeze	F value
Initially motility	Pure Jersey bulls	80.55±1.70^aA^	62.77±1.35^bA^	46.11±1.43^cA^	130.741**
	Crossbred Jersey bulls	80.00±1.80^aA^	65.00±1.66^bA^	47.22±1.08^cA^	11.781**
	F value	0.50^NS^	1.071^NS^	0.382^NS^	
Live sperm %	Pure Jersey bulls	83.63±1.08^aA^	71.72±1.09^bA^	58.67±1.02^cA^	136.585**
	Crossbred Jersey bulls	80.25±1.31^aA^	67.91±1.20^bB^	51.63±0.97^cB^	148.964**
	F value	3.955^NS^	5.429*	24.715**	
Abnormal sperm %	Pure Jersey bulls	8.38±0.32^aA^	12.30±0.39^bA^	16.75±0.42^cA^	118.587**
	Crossbred Jersey bulls	9.00±0.45^aA^	12.91±0.48^bA^	18.11±0.64^cA^	80.124**
	F value	1.169^NS^	0.038^NS^	3.091^NS^	
HOST %	Pure Jersey bulls	71.88±0.77^aA^	62.05±0.80^bA^	47.27±1.05^cA^	196.121**
	Crossbred Jersey bulls	72.77±1.02^aA^	62.11±0.89^bA^	45.94±1.33^cA^	150.798**
	F value	0.479^NS^	0.002^NS^	0.619^NS^	
Acrosome intact %	Pure Jersey bulls	89.05±0.83^aA^	81.33±0.71^bA^	71.94±0.86^cA^	112.743**
	Crossbred Jersey bulls	86.55±0.57^aB^	78.66±0.42^bB^	69.38±0.53^cB^	279.249**
	F-value	6.103*	10.352**	6.300*	
DNA intact %	Pure Jersey bulls	99.88±0.07^aA^	100.00±0.00^a^	99.94±0.05^bA^	13.300**
	Crossbred Jersey bulls	99.61±0.11^aA^	100.00±0.00^a^	99.44±0.53^bA^	13.163**
	F value	0.347^NS^		0.0405^NS^	

Mean bearing different superscript (A, B) between rows and (a, b, c) between column differ significantly.

* Indicate significant at 5% level,

** Indicate significantly at 1% and 5% level, NS=Non-significant, HOST=Hypoosmotic swelling test

### Live sperm percentage

The average live sperm percentage of neat semen in pure and crossbred Jersey bulls was 83.63±1.08% and 80.25±1.31% which did not differ significantly (p>0.05) between the breeds (Table-2). Live sperm percentage of both breeds at pre-freeze stage differed, significantly at 1% level from their neat semen values. Furthermore, it also differed significantly between breeds at 1% level at the pre-freeze stage. Live sperm percentage was found to be significantly (p<0.01) higher in pure Jersey bulls (71.72±1.09%) as compared to crossbred Jersey bulls (67.91±1.20%) (Table-2). The present findings in pure Jersey bulls were corroborates with Singh *et al*. [25]. However, Kumar [26] recorded higher live sperm percentage at post-equilibration than the present findings. In crossbred Jersey bulls, the live sperm percentage found to be lower than the values as reported by Sharma [27]. Live sperm percentage of both breeds at post-freeze stage differed significantly from their previous stages at 1% level (Table-2). Furthermore, the live sperm percentage recorded in pure Jersey bull (58.67±1.02%) is significantly higher (p<0.05) than crossbred Jersey bulls (51.63±0.97%). The present findings of live sperm percentage in pure Jersey bulls corroborate with the observation reported by Verma *et al*. [28], whereas Mandal *et al*. [29] recorded higher live sperm percentage as compare to present finding of pure Jersey bulls. Zodinsanya *et al*. [30] recorded lower live sperm percentage in pure bulls (Sahiwal, Jersey, and HF) as compare to present value to pure Jersey bulls. Vyas *et al*. [31] reported lower live sperm percentage in crossbred bulls as compare to present finding. Zodinsanya *et al*. [30] recorded higher live sperm percentage in crossbred bulls (HF, Red Dane, and Sahiwal) as compare to crossbred Jersey bulls. The statistical difference in live sperm percentage recorded among stages may be attributed to the deleterious effect of dilution, cooling, freezing, and thawing procedure. However, a statistical difference in breeds may be attributed to the breed differences. The variation in live sperm count may be due to the frequency of collection as reported by Tomar *et al*. [32], age of breeding bull and season as describe by Sharma *et al*. [33], extender used for dilution, cooling, and thawing process.

### Sperm abnormalities

No significant difference was observed in abnormal spermatozoa percentage between the breeds as their mean value was 8.38±0.32% and 9.00±0.45%, respectively (Table-2). The percentage increased significantly (p<0.01) at a pre-freeze stage in both breeds when compared with their neat semen value but no significant difference was observed between the breeds (Table-2). The sperm abnormalities in pure and crossbred Jersey bulls were having similar values as 12.30±0.39% and 12.91±0.48%. The abnormal sperm percentage increased significantly (p<0.01) further at the post-freeze stage in both breeds but no significant difference recorded between breeds (Table-2). The sperm abnormalities were higher in crossbred Jersey bulls (18.11±0.64%) as compared to pure Jersey bulls (16.75±0.42%). Mandal *et al*. [29], Zodinsanya *et al*. [30] recorded higher abnormal sperm as compared to the present findings recorded in pure Jersey bulls.

### HOST

The average mean values in pure and crossbred Jersey bulls were 71.88±0.77 and 72.77±1.02%. Statistical analysis revealed no significant difference (p>0.05) in the HOS positive spermatozoa in neat semen of breeds (Table-2). The findings for HOST % recorded in pure Jersey bulls were in close agreement with Martins *et al*. [34], Shukla *et al*. [35]. However, Zubair *et al*. [36] recorded lower HOST reacted spermatozoa than the present findings in pure Jersey bulls. The HOST % reduced significantly (p<0.01) at the pre-freeze stage from their neat semen values in both breeds, however, no significant difference was observed between the breeds. The average mean values in pure and crossbred Jersey bulls were 62.05±0.80% and 62.11±0.89%. The average mean values in pure and crossbred Jersey bulls at post-freeze were 47.27±1.05% and 45.94±1.33%; however, no significant differ between the breeds. The HOST reacted spermatozoa % also reduced significantly (p<0.01) further at post-freeze than earlier stages (Table-2). The differences in the percentage recorded may be due to mass activity, progressive motility as motility depends on transport of compounds across the membrane of spermatozoa [12], sperm count, total sperm with intact acrosome [37], and total sperm abnormalities in semen of different breeds [38]. The significant difference noted in the mean percentage of neat, pre-freeze, and post-freeze semen of both breeds is mainly due to cryopreservation. It is a non-physiological method that involves a high level of adaptation of biological cells to the osmotic and thermal shocks that occur during dilution, cooling-freezing and also during thawing procedures [39]. Damage occurs during-thawing procedures affect mainly the cellular membranes (plasma and mitochondrial) [40].

### Intact acrosome percent

The mean average value of neat semen in pure and crossbred Jersey bulls was 89.05±0.83 and 86.55±0.57%. There was significant (p<0.05) difference in the acrosome intactness percent of sperm between two breeds in neat semen. The acrosome percentage in pure Jersey bulls was in close agreement with that reported in bulls by Kumar [26]. However, it was higher as compared to Mishra *et al*. [41]. The lower acrosome intactness recorded by Nilani *et al*. [15] in Jersey bulls. In crossbred Jersey bulls, our present findings values are higher than the triple crossbred bulls [33]. There was a significant (p<0.01) difference in the percentage of acrosome integrity between breeds and also at pre-freeze stage (Table-2). The mean value in pure Jersey is higher (81.33±0.71%) than crossbred Jersey bulls (78.66±0.42%). The average pre-freeze acrosome integrity of sperm in pure Jersey bulls was in close agreement with that reported by Kumar [26]. In crossbred Jersey bulls, the acrosome intactness values was lower than the Jersey × local crossbred bulls reported by Sharma [27] in pre-freeze stage, and there was a significant difference between the breeds as well as the post-freeze stage at 5% level. The mean value in the post-freeze stage of pure Jersey is higher (71.94±0.86%) than crossbred Jersey bulls (69.38±0.53%) which differ significantly at 5% level. Whereas, Chowdhury *et al*. [42] reported higher acrosome integrity (75.15±0.71) in crossbred Jersey bulls as compared to pure Jersey bulls (73.05±0.059) at the post-freeze stage. Whereas, Zodinsanya *et al*. [30] recorded higher acrosome integrity percentage in pure bulls (Sahiwal, Jersey, and HF) as compare to present findings. The variation may be due to breeds, environments and cold shock, freezing technique, and staining technique. Decrease in acrosomal integrity was due to the damage caused to acrosome during dilution, cooling, freezing, and thawing process as describe by Tasseron *et al*. [43].

### DNA integrity

In the present research work, the DNA damage detected in a very few samples (range 0-1%) of neat semen in pure Jersey and crossbred Jersey. Thus, no significant difference (P>0.05) was observed regarding DNA damage in neat semen of both breeds (Table-2). The present findings in pure and crossbred Jersey bulls were in close agreement as described by Mondal *et al*. [44] in Mithun bulls. At pre-freeze stage, no DNA damage was observed. There was a significant difference (P<0.01) in post-freeze stages but no significant difference was observed between the breeds. In pre-freeze stage, our present values are similar to that of Awassi ram reported by Nur [45]. However, in post-freeze semen DNA integrity recorded for pure and crossbred Jersey bulls was in close agreement with that of Nellore bulls [46]. The present values of DNA integrity are higher as compared to Mithun bulls in post-freeze stage [44]. The freezing-thawing procedures cause the deterioration of the DNA integrity [45]. The process of cryopreservation is known to cause more production of reactive oxygen species, as antioxidant defenses are reduced in the process which is responsible for DNA damage [47].

## Conclusion

The ejaculate volume, color, consistency, sperm concentration, and initial motility in pure and crossbred Jersey bulls did not differ. Mass activity, live sperm percentage, and acrosome integrity were significantly higher in pure Jersey bulls as compared to crossbred Jersey bulls. No statistical difference was observed in abnormal sperm; HOS reacted spermatozoa and DNA integrity percentage between breeds. Significant decrease in the motility, live sperm percentage, HOS reacted spermatozoa, acrosome integrity and significant increase in abnormal count from neat to pre-freeze and from pre-freeze to post-freeze semen of pure and crossbred Jersey bulls was due to the deleterious effect of cooling-freezing and thawing procedures. The semen quality of pure Jersey bull semen was better than the crossbred bull. It may be concluded that the estimation of live sperm percentage and acrosome integrity is useful in the assessment of semen quality in bulls.

## Authors’ Contributions

UK collected semen sample from each bull and processed as per standard protocol, analyzed the data and APG design the work and help in preparing the final manuscript. SKS, MSP, and SWB help in determining DNA damages and PLB, AJP, and BRR help me in semen evaluation in Frozen Semen Laboratory. CKL help me counting slides and searching research articles. All authors read and approved the final manuscript.
